# Stage of infection with *Mycobacterium avium* subsp. *paratuberculosis* impacts expression of Rab5, Rab7, and CYP27B1 in macrophages within the ileum of naturally infected cows

**DOI:** 10.3389/fvets.2023.1117591

**Published:** 2023-02-03

**Authors:** Taylor L. T. Wherry, Mark Heggen, Adrienne L. Shircliff, Shankumar Mooyottu, Judith R. Stabel

**Affiliations:** ^1^Department of Veterinary Pathology, College of Veterinary Medicine, Iowa State University, Ames, IA, United States; ^2^United States Department of Agriculture-Agricultural Research Service (USDA-ARS), National Animal Disease Center, Ames, IA, United States; ^3^Department of Nutritional Sciences, College of Agricultural and Life Sciences, University of Wisconsin-Madison, Madison, WI, United States

**Keywords:** *Mycobacterium avium* subsp. *paratuberculosis*, macrophage, Rab5, Rab7, vitamin D, CYP27B1, CYP24A1, bovine

## Abstract

**Introduction:**

Macrophages are the preferential target of *Mycobacterium avium subsp. paratuberculosis* (MAP), the etiologic agent of ruminant paratuberculosis. Uptake of pathogens by intestinal macrophages results in their trafficking through endosomal compartments, ultimately leading to fusion with an acidic lysosome to destroy the pathogen. MAP possesses virulence factors which disrupt these endosomal pathways. Additionally, levels of serum vitamin D_3_ have proven relevant to host immunity. Dynamics of endosomal trafficking and vitamin D_3_ metabolism have been largely unexplored in bovine paratuberculosis.

**Methods:**

This study aimed to characterize expression of early and late endosomal markers Rab5 and Rab7, respectively, within CD68+ macrophages in frozen mid-ileum sections harvested from cows at different stages of natural paratuberculosis infection. Additionally, factors of vitamin D_3_ signaling and metabolism were characterized through expression of vitamin D_3_ activating enzyme 1α-hydroxylase (CYP27B1), vitamin D_3_ inactivating enzyme 24-hydroxylase (CYP24A1), and vitamin D_3_ receptor (VDR) within CD68+ ileal macrophages.

**Results and discussion:**

Cows with clinical paratuberculosis had significantly greater macrophage and MAP burden overall, as well as intracellular MAP. Total expression of Rab5 within macrophages was reduced in clinical cows; however, Rab5 and MAP colocalization was significantly greater in this group. Intracellular Rab7 colocalization with MAP was not detected in subclinical or Johne's Disease negative (JD-) control cows but was present in clinical cows. Additionally, macrophage CYP27B1 expression was significantly reduced in clinical cows. Taken together, the results from this study show disparate patterns of expression for key mediators in intracellular MAP trafficking and vitamin D metabolism for cows at different stages of paratuberculosis.

## 1. Introduction

Macrophages are key phagocytic cells that serve as a reservoir for *Mycobacterium avium* subsp. *paratuberculosis* (MAP) infection in ruminants, commonly known as Johne's disease (JD). Uptake of MAP by macrophages leads to a series of mechanisms which cultivate an environment to promote MAP persistence and dissemination within the host. Initial innate defense mechanisms traffic MAP within a phagosome through the endosomal pathway. However, *in vitro* studies have shown MAP can arrest phagosomal maturation and pathogen destruction by blocking recruitment of endosomal trafficking markers, such as late endosome lysosome associated membrane protein 1 (LAMP-1) and LAMP-2 (1, 2). In related species *Mycobacterium tuberculosis* (*M. tb*), the cell wall component mannosylated lipoarabinomannan (ManLAM) inhibits signaling mediators that play a direct role in phagosomal acquisition of Rab5 from early endosomes, ultimately facilitating disruption of events leading to bacterial destruction (3–5). By creating this interference, *M. tb* can successfully prevent the acquisition of downstream effectors necessary to direct fusion of the phagosome with lysosomes. Additional *M. tb* virulence factors directly inhibit late endosomal marker Rab7 recruitment to the phagosome, further ensuring pathogen survival (6).

Efforts to further characterize mechanisms of immune evasion by MAP has led our group to investigate impacts of a key nutritional factor, vitamin D_3_. Traditionally a fundamental regulator of calcium homeostasis, vitamin D_3_ also modulates immune responses important in prevention and resolution of infectious disease. This has been well-documented in human tuberculosis (7, 8), and more recently in bovine mastitis (9–11). Previous work in our lab has shown cows with clinical paratuberculosis have significantly reduced circulating 25-hydroxyvitamin D_3_ [25(OH)D_3_] levels compared to subclinical cows and JD- controls (12, 13), highlighting the potential importance of this nutritional deficit in severe bovine paratuberculosis.

Biological effects of vitamin D_3_ are exerted by the active metabolite 1,25-dihydroxyvitamin D_3_ [1,25(OH)_2_D_3_]. Conversion of 25(OH)D_3_ to 1,25(OH)_2_D_3_ is accomplished through the action of 1α-hydroxylase (CYP27B1), whose expression is found in multiple immune cell types including monocytes/macrophages, dendritic cells, and T helper cells (14–17). Currently, the most detailed mechanism of 1,25(OH)_2_D_3_ signaling begins with binding of 1,25(OH)_2_D_3_ to vitamin D receptor (VDR), mainly localized in the nucleus, where coupling with retinoid X receptor (RXR) occurs (18). This complex ultimately binds with target genes to alter their expression. Membrane signaling through VDR and other receptors, such as protein disulphide isomerase family A member 3 (PDIA3), resulting in non-genomic vitamin D effects through the NF-κB and STAT3 pathways have been postulated (19).

The objective of this study was to characterize expression of early (Rab5) and late (Rab7) endosomal trafficking markers, along with vitamin D elements CYP24A1, CYP27B1, and VDR in macrophages from cows at subclinical and clinical stages of paratuberculosis using fluorescent labeling of frozen mid-ileum tissue sections and confocal microscopy. To the best of our knowledge, this is the first study to investigate these endosomal trafficking markers and vitamin D hydroxylases in intestinal tissue macrophages from cattle with paratuberculosis. Mechanisms of immune dysfunction in MAP infection remain to be fully elucidated and the results of this study provide valuable insights into the signaling dynamics between MAP and macrophages within intestinal tissue.

## 2. Materials and methods

### 2.1. Animals

Holstein dairy cows aged 3–15 years were used in this study. To prevent cross-contamination, all animals were housed separately on-site according to positive or negative infection status with MAP. All experimental procedures were approved by the IACUC (National Animal Disease Center, Ames, IA). Housing facilities are accredited by the American Association for Accreditation of Laboratory Animal Care.

Cows were stratified into infection status groups utilizing diagnostic tests measuring serum MAP-specific antibody levels (Herdchek; IDEXX, Westbrook, ME), bovine IFN-γ plasma levels (Bovigam; Prionics, La Vista, NE), and fecal shedding detected by culture on Herrold's egg yolk medium (Becton Dickinson, Sparks, MD) as previously described (20). Clinical cows (*n* = 10) had an average age of 5 years and were ELISA positive for MAP serum antibody, with an average S/P ratio of 2.44. The average MAP-specific IFN-γ recall response for this group was OD_450_ 0.50 ± 0.35 (Abs_450nm_MPS–Abs_450nm_NS). Most of these animals were also culture positive for MAP, having an average of ~138 CFU/g fecal matter. Subclinical cows (*n* = 10) had an average group age of 8 years and were mostly ELISA negative for MAP serum antibodies, with one subclinical cow weakly positive having an S/P of 0.91. The subclinical group had an average IFN-γ OD_450_ of 0.16 ± 0.09. Two subclinical cows were fecal culture positive for MAP and had an average shedding value of 1.5 CFU/g fecal matter. Control cows (*n* = 11), averaging 8 years of age, were negative for all diagnostic tests.

### 2.2. Tissue collection and processing

Samples of mid-ileal tissue were collected at necropsy and rinsed with Dulbecco's phosphate buffered saline (D-PBS) at pH 7.4 (Sigma-Aldrich, St. Louis, MO). The luminal side was placed on top of a liver section with a thin layer of Tissue-Tek optimum cutting temperature (OCT) compound (Sakura Finetek, Torrance, CA) interface to protect microvilli structure from mechanical and environmental disruption. This also aided in determining tissue orientation upon cryosectioning. Tissue samples were then wrapped in aluminum foil and snap-frozen by placement in a tin cup of cold isopentane (Sigma-Aldrich) housed within a small cooler of dry ice and 95% ethanol slurry for a minimum of 5 min, then wrapped again in aluminum foil and transferred on dry ice to be stored at −80°C.

When cryosectioning was ready to be performed, tissue sections were removed from storage at −80°C and placed in a cryostat to acclimate to −20°C for at least 30 min. The entire tissue sample was embedded within Tissue-Tek optimal cutting temperature (O.C.T.; Sakura Finetek, Torrance, CA) and cut in 6 μm sections, then adhered to MAS slides (Matsunami Glass, Bellingham, WA). The tissue sections were dried overnight at room temperature and fixed in a 50% acetone/50% methanol solution for 5 min. Slides were stored at −80°C until ready for further processing.

### 2.3. Immunofluorescence

Prior to beginning the tissue immunofluorescence (IF) protocol, CYP27B1 antibody (PA5-79128; Invitrogen, Carlsbad, CA) had its stock buffer exchanged to remove bovine serum albumin (BSA) using the Pierce Antibody Cleanup Kit (44600; Thermo Scientific, Rockford, IL). Buffer exchanged antibody was transferred to a 30 K molecular weight cutoff Pierce concentrator microcentrifuge column (88529; Thermo Scientific) and centrifuged at 12,000 × g in 30 s increments to a final volume of 100 μl. The 100 μl of antibody was conjugated using the AF594 Lightning-Link conjugation kit (ab269822; Abcam, Waltham, MA) according to the manufacturer's instructions. Additionally, Rab5 primary antibody was directly conjugated to AF594 using the same Lightning-Link conjugation kit.

Primary antibodies used in this study were split into multiple protocols due to our constrains of four lasers. All antibodies were diluted in 0.05M Tris buffer and incubated for 1 h each at room temperature in a humidified chamber protected from light. The tissue underwent 3 wash steps with 0.05 M Tris/0.2% Tween20/0.9% NaCl (Tris/Tween buffer) following incubation with each antibody.

Tissue sections were removed from storage at −80°C and allowed to equilibrate to room temperature. A thin hydrophobic layer was drawn around each tissue section using a PAP pen (Life Technologies, Carlsbad, CA) to keep liquid components localized. Tissue was rehydrated with 0.05 M Tris buffer for 10 minutes, followed by addition of 3,3′-diaminobenzidine (DAB) (Vector Labs, Burlingame, CA) for 10 min in a dark, humidified chamber to quench autofluorescence. Slides were then washed 3 times alternating with 0.05 M Tris buffer and Tris/Tween buffer. Non-specific labeling was blocked for 30 min using serum-free blocking buffer (X0909, Agilent Technologies, Santa Clara, CA). Primary and secondary antibodies were added following this step and are detailed in the individual protocols below.

CYP27B1 and CYP24A1 antibodies were paired together, and this protocol began with labeling of ileal tissue macrophages with CD68 primary antibody (1:300, M0718; Agilent Technologies), followed by labeling with an AF647 conjugated secondary antibody (1:750, 115-605-205; Jackson Labs). Next, the CYP24A1 (1:25, LS-C407760; LSBio, Seattle, WA) primary antibody was added, followed by AF488 secondary antibody (1:250, A11070; Invitrogen). Lastly, CYP27B1 was labeled in the tissue using a primary antibody directly conjugated to AF594 (1:40, PA5-79128; Invitrogen).

Detection of Rab5 began with addition of CD68 (1:300, Agilent Technologies) and MAP (1:1,000, #272 in-house, NADC) primary antibodies to the tissue concurrently in a cocktail. Secondary antibodies conjugated to AF647 (115-605-205; Jackson Labs) or AF488 (1:1,000, A11070; Invitrogen) were also added together and were used to detect each primary antibody, respectively. Rab5 (1:40, MBS612620; MyBioSource) directly conjugated to AF594 was added to the tissue last.

The Rab7 protocol also began with labeling of tissue macrophages and MAP through addition of CD68 (1:300, Agilent Technologies) and MAP (1:1,000, #272 in-house, NADC) primary antibody cocktail. Secondary antibodies were also added in a cocktail, with CD68 being detected by AF647 (1:1,000, 115-605-205; Jackson Labs) and MAP detected by AF488 (1:1,000, A11070; Invitrogen). Rab7 primary (1:100, ab50533; Abcam) was then incubated with the tissue, followed by an AF594 secondary antibody (1:250, A21145, Invitrogen).

The Vitamin D receptor (VDR) protocol began with the labeling of CD68 (1:300, Agilent Technologies) followed by addition of AF647 secondary antibody (1:750, 115-605-205; Jackson Labs). The VDR primary antibody (1:10, LS-C407668; LSBio) was then added, followed by labeling with an AF594 secondary antibody (1:250, A11072, Invitrogen).

Lastly, for all protocols counterstaining was achieved using DAPI diluted in 0.05 M Tris buffer to a concentration of 1μg/ml and incubated with the tissue for 10 min. Slides underwent one more wash step with 0.05 M Tris buffer prior to addition of VECTASHIELD Vibrance mounting media (Vector Labs) and coverslipping with 24 × 30 mm Richard-Allan Scientific Slip-Rite #1.5 (Thermo Scientific, Carlsbad, CA). Mounting media was allowed to cure overnight at room temperature in the dark prior to imaging.

### 2.4. Histochemistry

Prior to histochemical staining, all slides were equilibrated to room temperature and rehydrated in deionized water for 5 min. Sequential slides corresponding with IF protocols were stained using Ziehl-Neelsen acid fast (AF) and Harris' hematoxylin and eosin (H&E) methods to detect acid-fast mycobacteria and assess tissue morphology, respectively.

Slides that underwent AF staining were incubated for 1 h in carbol fuchsin solution at room temperature. Stock acid alcohol was used to decolorize tissue for ~10 s, which resulted in the tissue obtaining a pale pink color. Slides were then washed in a running water bath for 10 min, followed by a 5 min counterstain with Harris' hematoxylin in a Leica autostainer (Leica Biosystems, Buffalo Grove, IL). Lastly, slides were rinsed again in a running water bath and coverslipped using xylene substitute mountant (Epredia, Portsmouth, NH).

H&E staining was also performed in a Leica autostainer beginning with incubation for 5 min in Harris' hematoxylin to label nuclei, followed by rinsing in deionized water for 5 min. Slides were then dipped in acid alcohol and rinsed in a deionized water bath for 5 min, then incubated for 1 min in 70% alcohol. To stain cytoplasm, slides were incubated with eosin for ~15 s. The slides were dehydrated by dipping twice in 95% alcohol and three times in 100% alcohol, each for 1 min. Lastly, slides were cleared of OCT by dipping three times in Pro-Par for 5 min each prior to coverslipping using xylene substitute mountant (Epredia, Portsmouth, NH).

A subset of cows from the CYP27B1/CYP24A1 protocol (Set 1) were used for the Rab5, Rab7, and VDR protocols (Set 2) due to tissue availability. New tissue sample cryostat chucks were made for this subset of cows, and concurrent AF and H&E slides were stained for pathology analysis.

### 2.5. Tissue pathology

Tissue lesion severity and presence of MAP was evaluated histologically by a board certified veterinary pathologist. Tissue slides were examined at 200 × and 20 fields were read each. MAP burden and granuloma severity were scored within a range of 0–5, with methods adopted from Palmer et al. (21) and Stabel et al. (22) and used previously in our lab (23–25). Briefly, a score of 0 indicated no MAP/granuloma presence and a score of 1 represented scarce MAP and granulomas within each tissue section. Increasing scores correlate with increasing MAP burden and granuloma severity, with a score of 5 representing a high amount of MAP present and severe granulomas disrupting most of the tissue architecture.

### 2.6. Confocal microscopy

Images were acquired with a 60 × Nikon Plan Apochromat lambda objective with 1.4 numerical aperture using oil immersion and 6.2 s pixel dwell time on a Nikon A1 Resonance Plus confocal microscope using NIS-Elements Advanced Research software v5.21 (Nikon, Melville, NY). The instrument contains a four-laser gallium-arsenide-phosphide/normal photomultiplier tube (GaAsP PMT) fluorescence detector unit (A1-DU4) with two GaAsP PMTs (488 and 561 nm) and two normal PMTs (405 and 640). Fluorescent signal was detected sequentially using the following solid-state diode lasers and bandpass filters: 405 nm (450/50 nm), 488 nm (525/50 nm), 561 nm (600/50 nm), and 640 nm (685/70 nm). A minimum of 10 images were acquired per cow.

Following image acquisition, binary layers for each laser channel were created with thresholds established from control slides to exclude non-specific background. The average area (μm^2^) expressing fluorescence signal for each marker was measured per image field. All analyses were run on unaltered images and representative images chosen for this manuscript have had lookup tables applied within the Nikon NIS-Elements software to brighten signal and were post-processed in Adobe Photoshop (version 22.0; San Jose, CA) to further increase color brightness for printing purposes. All alterations were uniformly applied to the entire image.

### 2.7. Statistics

Statistical analysis was performed using R Statistical Software (version 4.0.3, R Foundation for Statistical Computing, Vienna, Austria) and RStudio (version 1.3.1093, Boston, MA). Data measuring total amount of macrophages present and MAP burden were aggregated from separate IHC protocols performed and analyzed using the mixed model function “lme” from package “nlme” (26) to account for repeat observations. Statistical models for all other confocal data sets were analyzed by ANOVA using the linear model function “lm” (27) and all *post-hoc* tests were performed using the package “emmeans” (28) with a Tukey adjustment for multiple comparisons. Transformations of the data by log or log (1 + x) were performed where standardized residuals were not normally distributed. Pathology assessment scores were analyzed by using the Fisher's exact test with two-sided probability (*Pr* < 0.05) through function “fisher.test” from package “stats” in base R (27). Pearson correlations were performed using function “rcor.test” from package “ltm” and a multiple comparisons correction was applied using the Holm-Bonferroni method (29).

## 3. Results

### 3.1. Tissue macrophages and pathologic assessment

**Figure 1:**
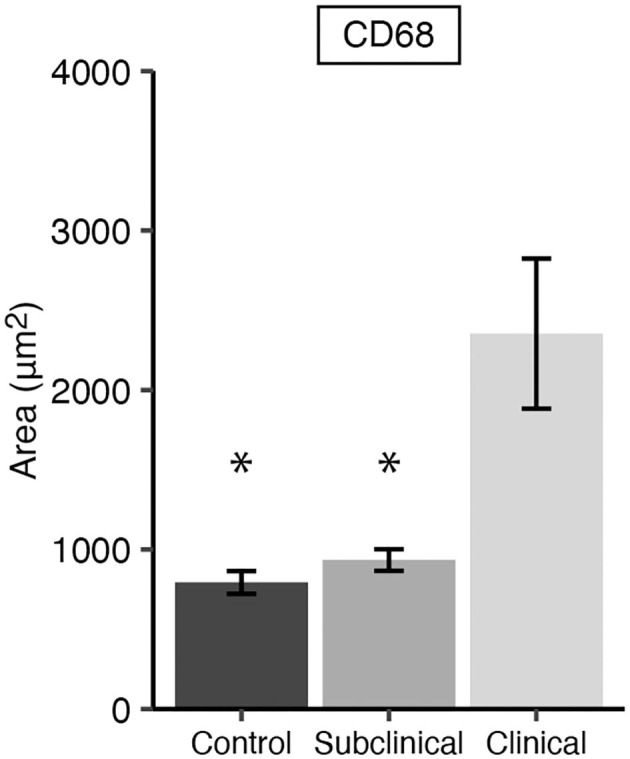
CD68 expression representing total macrophages present in frozen mid-ileal tissue from dairy cows naturally infected with *Mycobacterium avium* subsp. *paratuberculosis* (MAP) (clinical *n* = 9, subclinical *n* = 9) or JD- controls (*n* = 8). Data are presented as the mean fluorescence area μm^2^ ± SE and significance is indicated as * < 0.05 as compared to the clinical group.

Immunofluorescent assessment of CD68+ macrophages in the mid-ileum tissue from each cow showed animals in the clinical stage of infection had significantly greater numbers compared to subclinical (Figure 1; *P* < 0.05) and JD- cows (*P* < 0.05). No significant difference in the number of macrophages were observed between JD- control and subclinical animals.

Histologically present granulomatous inflammation was scored for each JD status group (Table 1). Subclinically infected cows showed no granulomatous lesions; however, one subclinical cow had a small focus of epithelioid macrophage aggregates, but no AF bacteria were present. All clinical cows had granulomatous lesions present. A significant association between subclinical/clinical JD status and granulomatous inflammation score was shown through Fisher's Exact Test (*P* < 0.001; Table 1).

**Table 1:** Frozen bovine ileum pathology assessment^a^.

		**AF score**						**AF location**			**Granulomatous inflammation score**					
	**Stage of JD**	**0**	**1**	**2**	**3**	**4**	**5**	**NA**	**LP**	**LP/SM**	**0**	**1**	**2**	**3**	**4**	**5**
	JD- control	10	0	0	0	0	0	10	0	0	10	0	0	0	0	0
	Subclinical	10	0	0	0	0	0	10	0	0	10	0	0	0	0	0
Set *1^*b*^*	Clinical	2	0	2	5	0	1	0	7	3	0	0	4	4	1	1
	Fisher's exact test															
	Clinical-subclinical	*P* < 0.001						*P* < 0.001			*P* < 0.001					
	JD- control	8	0	0	0	0	0	8	0	0	8	0	0	0	0	0
	Subclinical	9	0	0	0	0	0	8	0	0	9	0	0	0	0	0
Set 2	Clinical	3	0	3	0	2	0	0	6	2	1	2	3	1	2	0
	Fisher's exact test															
	Clinical-subclinical	*P* < 0.01						*P* < 0.001			*P* < 0.001					

AF, acid fast; LP, lamina propria; LP/SM, lamina propria/submucosa; NA, not applicable.

^a^Frequency counts of cows with different AF scores, AF locations, and granulomatous inflammation scores.

^b^Set 1 represents the group of cows used for the CYP27B1/CYP24A1 IF protocol. Set 2 represents the group of cows used for the Rab5, Rab7, and VDR IF protocols.

### 3.2. MAP burden

**Figure 2:**
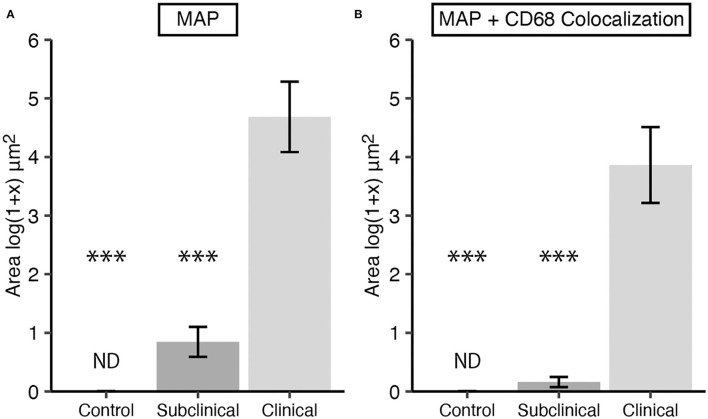
*Mycobacterium avium* subsp. *paratuberculosis* (MAP) burden in frozen sections of mid-ileal tissue from dairy cows. Animals were stratified into groups by natural MAP infection (clinical *n* = 9, subclinical *n* = 9) or JD- controls (*n* = 8). **(A)** Total MAP and **(B)** MAP within macrophages, defined as MAP colocalization with CD68, were measured. Data are presented as the mean fluorescence area [log(1 + x) μm^2^] ± SE and significance is indicated as *** < 0.001 as compared to the clinical group. Non-detected measurements are indicated by ND.

Total MAP burden and intracellular MAP within macrophages was also measured by IF. Cows in clinical stage disease had significantly greater (Figure 2A; *P* < 0.001) overall MAP burden compared to JD- control and subclinically infected animals. No significant difference in the overall number of MAP between JD- controls and subclinicals was observed; however, no MAP was detected in JD- control cows and ~66% of subclinical cow tissue samples had detectable MAP. Clinical cow tissue samples had a MAP detection rate of 100%. Additionally, clinical cows had greater intracellular MAP than subclinical cows and JD- controls as measured by MAP colocalization with CD68 (Figure 2B; *P* **<** 0.001).

Histochemical analysis of MAP burden was performed through AF staining. Aligning with the confocal IF data, clinical cows had the greatest amount of MAP present in the ileum. The presence of MAP was not detected by AF stain in tissues from subclinical or JD- control cows. Additionally, Fisher's Exact Test revealed higher AF scores were associated with the clinical group for both set 1 (*P* < 0.001; Table 1) and set 2 protocols (*P* < 0.01) when compared to the subclinical group. MAP was largely found in the lamina propria, and some animals observed bacilli extending from the lamina propria into the submucosa (Table 1). Furthermore, a strong, positive correlation was observed between AF and granulomatous inflammation scores (*r* = 0.935, *P* < 0.001). Positive correlations were also observed between the amount of macrophages detected by IF and both AF score (*r* = 0.679, *P* < 0.01) and granulomatous inflammation score (*r* = 0.602, *P* < 0.05).

### 3.3. Endosomal marker expression within macrophages

Early and late endosomal markers Rab5 and Rab7 were investigated by IF and measured through confocal microscopy. Total expression of each endosomal marker was measured by its colocalization with CD68, with and without colocalization with MAP.

**Figure 3:**
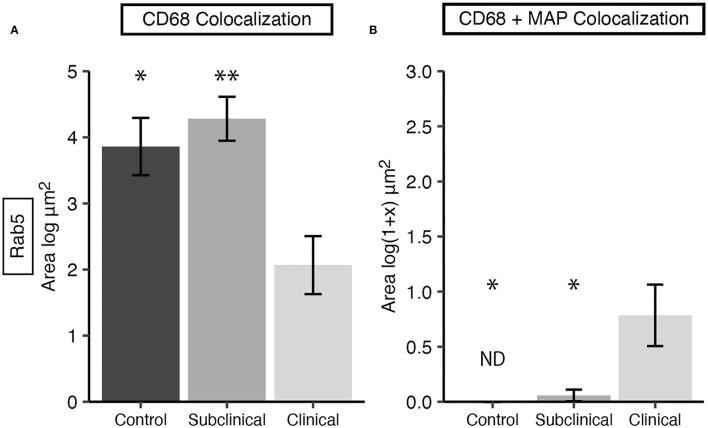
Early endosomal marker Rab5 expression in frozen tissue sections from bovine mid-ileum. Cows were grouped according to stage of paratuberculosis infection (clinical *n* = 9, subclinical *n* = 9, JD- control *n* = 8). **(A)** Rab5 expressed within CD68+ macrophages, presented as the mean fluorescence area (log μm^2^) ± SE. **(B)** Rab5 and MAP colocalization within CD68+ macrophages, presented as the mean fluorescence area [log(1 + x) μm^2^] ± SE. Significance is indicated as * < 0.05 and ** < 0.01 compared to the clinical group and non-detected measurements are indicated by ND.

**Figure 4:**
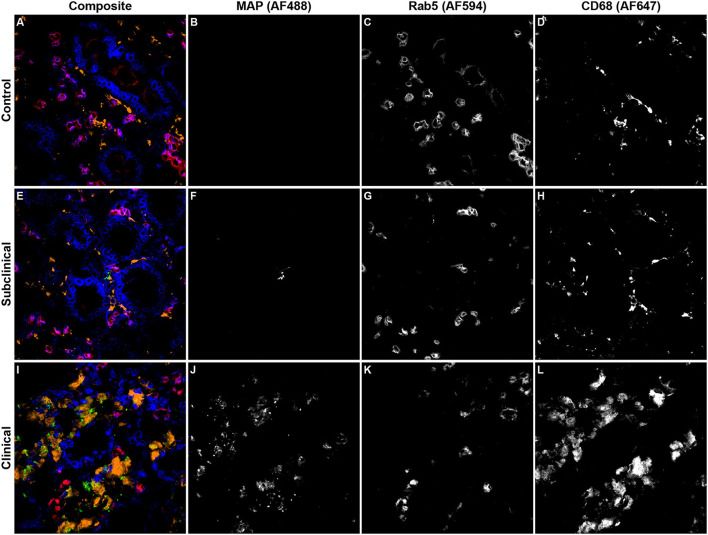
Representative confocal microscopy images of frozen mid-ileum sections from JD- control cows **(A–D)** and cows in subclinical **(E–H)** or clinical **(I–L)** stages of paratuberculosis. Composite images overlaying each laser channel are presented in **(A, E, I)**. *Mycobacterium avium* subsp. *paratuberculosis* (MAP; green) labeling was detected using an AF488 secondary antibody and excited on the 488 nm laser **(B, F, J)**, while Rab5 (red) primary antibody was directly conjugated to AF594 and excited on the 561 nm laser **(C, G, K)**. CD68 (orange) was labeled with an AF647 secondary antibody and excited on the 641 nm laser **(D, H, L)**. Counterstaining of nuclei (blue) was achieved using DAPI and was excited on the 405 nm laser.

Expression of Rab5 within tissue macrophages was shown to be significantly reduced in clinical cows compared to both subclinicals (Figure 3A; *P* < 0.01) and JD- controls (*P* **<** 0.05). No significant differences were observed between subclinical and JD- control animals. In contrast, Rab5 colocalization with intracellular MAP was significantly higher for clinical cows compared to JD- control (Figure 3B; *P* < 0.05) and subclinical cows (*P* < 0.05). Fluorescent expression of Rab5, MAP, and macrophages within the bovine ileum from a representative healthy (Figures 4A–D), subclinical (Figures 4E–H), and clinical cow (Figures 4I–L) are shown in a composite confocal microscopy image. This figure also shows visual representation of the increased MAP burden between subclinical and clinical cows.

**Figure 5:**
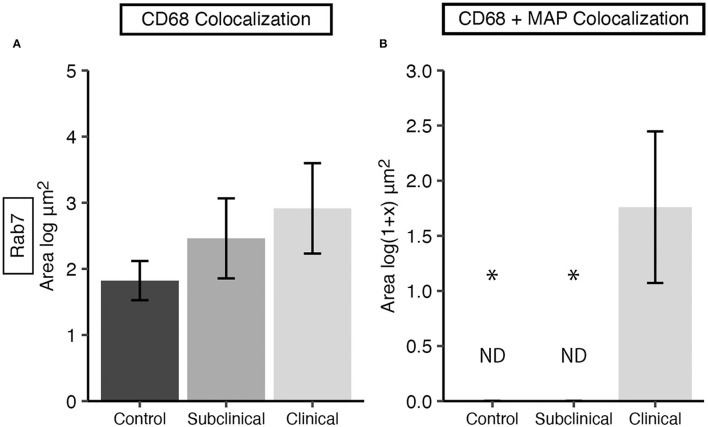
Late endosomal marker Rab7 expression in frozen tissue sections from bovine mid-ileum. Cows were grouped according to stage of paratuberculosis infection (clinical *n* = 9, subclinical *n* = 9, JD- control *n* = 8). **(A)** Rab7 expressed within CD68+ macrophages presented as the mean fluorescence area (log μm^2^) ± SE. **(B)** Rab7 and MAP colocalization within CD68+ macrophages presented as the mean fluorescence area [log(1 + x) μm^2^] ± SE. Significance is indicated as * < 0.05 compared to the clinical group and non-detected measurement is indicated by ND.

**Figure 6:**
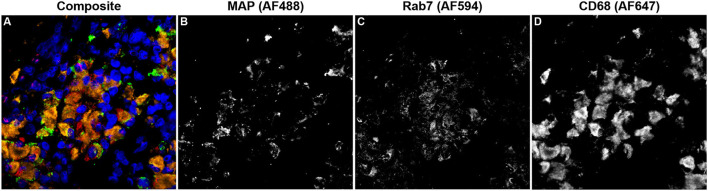
Confocal microscopy image of frozen mid-ileum tissue section from a cow with clinical stage paratuberculosis. The composite image **(A)** shows *Mycobacterium avium* subsp. *paratuberculosis* (MAP; green) detected using an AF488 secondary antibody and excited on the 488 nm laser **(B)**. Rab7 (red) expression was detected through excitation of an AF594 secondary antibody on the 561 nm laser **(C)**. CD68 (orange) was labeled with an AF647 secondary antibody and excited on the 641 nm laser **(D)**. Nuclei (blue) were counterstained using DAPI which was excited on the 405 nm laser.

Rab7 expression within tissue macrophages was not different among infection status groups (Figure 5A); however, clinical cows had significantly greater colocalization of Rab7 with MAP within tissue macrophages when compared to subclinical cows and JD- controls (Figure 5B; *P* < 0.05). Notably, no Rab7 colocalization was detected with intracellular MAP from subclinical cows. Fluorescent expression of Rab7, MAP, and macrophages within the bovine mid-ileum is shown in a confocal microscopy image from a representative clinical cow (Figures 6A–D).

### 3.4. CYP and VDR expression within macrophages

**Figure 7:**
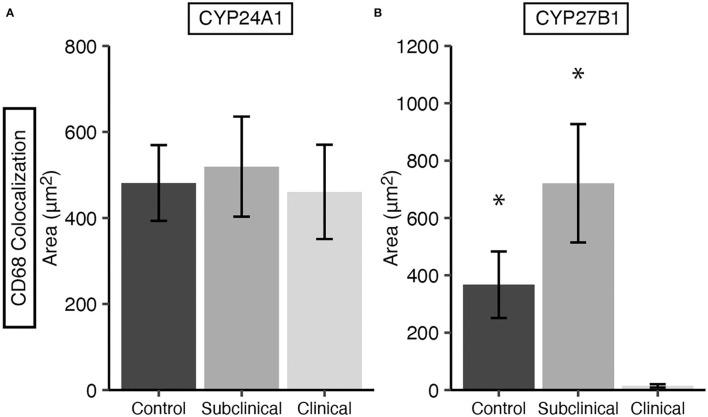
CYP24A1 **(A)** and CYP27B1 **(B)** expression within macrophages in frozen tissue sections from bovine mid-ileum. Expression was measured by CYP colocalization with CD68. Cows were stratified into groups by natural MAP infection (clinical *n* = 10, subclinical *n* = 10) or JD- controls (*n* = 10). Data are presented as the mean fluorescence area μm^2^ ± SE. Significance is indicated as * < 0.05 compared to the clinical group.

**Figure 8:**
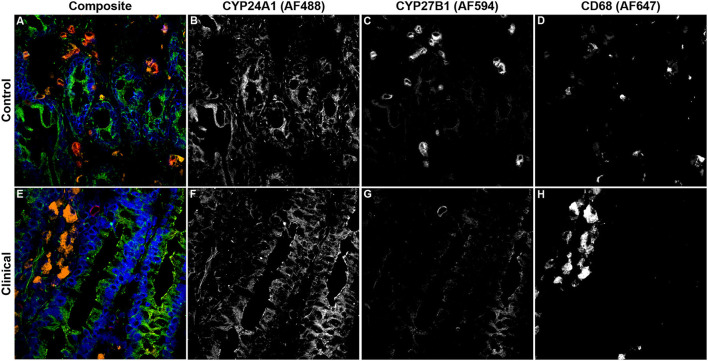
Confocal microscopy images of frozen mid-ileum sections from a JD- control cow **(A–D)** and clinical cow **(E–H)**. Composite images overlaying each laser channel are presented in **(A, E)**. CYP24A1 (green) labeling was detected using an AF488 secondary antibody and excited on the 488 nm laser **(B, F)**, while CYP27B1 (red) primary antibody was directly conjugated to AF594 and excited on the 561 nm laser **(C, G)**. CD68 (orange) was labeled with an AF647 secondary antibody and excited on the 641 nm laser **(D, H)**. Nuclei (blue) were counterstained using DAPI which was excited on the 405 nm laser.

Expression levels of vitamin D hydroxylases CYP24A1 and CYP27B1 were measured within the bovine ileum for each group of cows. CYP24A1 showed no significant level of variation among infection status groups (Figure 7A). In contrast, CYP27B1 expression was significantly reduced in clinical cows compared to JD- controls (Figure 7B; *P* < 0.05) and subclinical cows (*P* < 0.05). A representative confocal microscopy image from a JD- control (Figures 8A–D) and clinical cow (Figures 8E–H) show a high degree of CYP24A1 labeling in the enterocytes of the intestinal villi. CYP27B1 expression is also expressed in the villi enterocytes albeit in lower amounts. Furthermore, CYP27B1 expression was observed to colocalize with CD68+ macrophages. A high level of expression was also observed in another undetermined cell type which appears to dimly label with CD68 or not at all.

**Figure 9:**
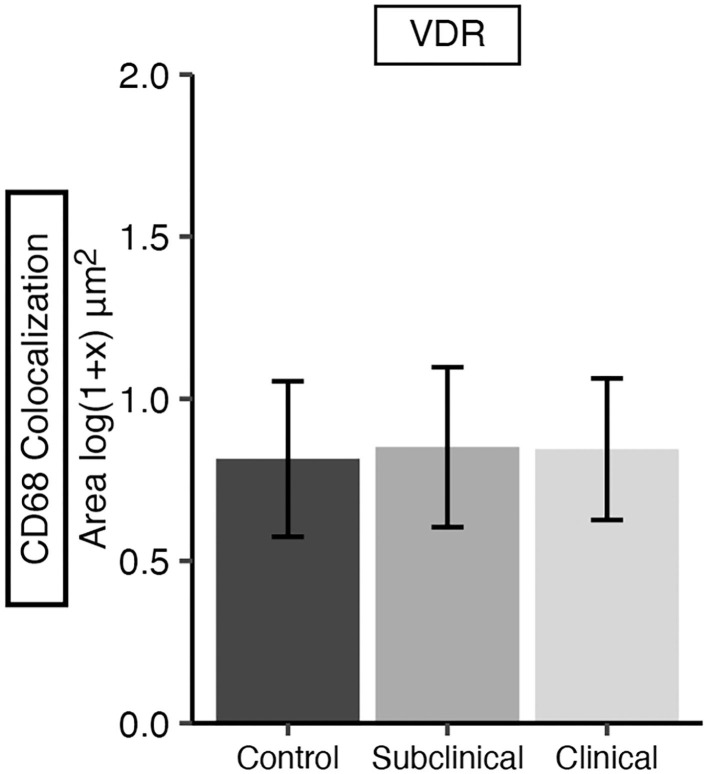
Vitamin D receptor (VDR) expression in frozen tissue sections from bovine mid-ileum. Cows were grouped according to stage of paratuberculosis infection (clinical *n* = 9, subclinical *n* = 9, JD- control *n* = 8). VDR expression was measured within CD68+ macrophages and is presented as the mean fluorescence area [log(1 + x) μm^2^] ± SE. No significant differences were observed among infection status groups.

**Figure 10:**
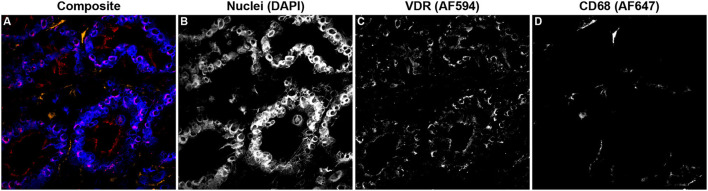
Confocal microscopy image of frozen mid-ileum tissue section from a cow with clinical stage paratuberculosis. The composite image overlaying each laser channel is presented in **(A)**. Nuclei (blue) were counterstained using DAPI which was excited on the 405 nm laser **(B)**. VDR (red) expression detected through excitation of an AF594 secondary antibody on the 561 nm laser **(C)** and shows purple where colocalized with nuclei. CD68 (orange) was labeled with an AF647 secondary antibody and excited on the 641 nm laser **(D)**.

Additionally, VDR expression in the mid-ileum was investigated which revealed no variation in expression among infection status groups (Figure 9). A fluorescent microscopy image from a representative clinical cow shows VDR labeling highly concentrated in the nuclei of enterocytes, along with lower levels of expression in CD68+ macrophages (Figures 10A–D).

## 4. Discussion

To maintain the chronic subclinical disease state, MAP has evolved niche survival mechanisms which allow it to manipulate normal machinery within the host macrophage. Many of these immune evasion tactics are largely yet to be fully understood, as a large obstacle in understanding MAP pathogenesis is parsing out which immune signaling responses benefit the host or pathogen at each stage of disease. The subclinical stage of MAP infection is associated with pro-inflammatory T helper 1 (Th1) cytokine responses largely dominated by IFN-γ, which direct host immune cells to attack and destroy the invading pathogen (30). Cows in this stage experience chronic infection lasting years and can intermittently shed MAP in their feces (31). A small fraction of animals will progress to the clinical stage, where MAP burden is high and a switch to a humoral T helper 2 (Th2) cytokine expression profile occurs, promoting antibody production (32). Unfortunately, these antibodies do little to contribute to disease resolution.

Focal granulomatous lesions containing fewer MAP are associated with subclinically infected cows, and the macrophages present skew toward a pro-inflammatory M1 host defense phenotype (33). In contrast, diffuse multibacillary lesions are commonly associated with clinical cows, and the macrophages present are predominantly of the anti-inflammatory M2 resolution and repair phenotype (33). As an animal experiences progressive disease severity as a result of increasing MAP burden, a concomitant increase in macrophage recruitment is observed (24). Cellular infiltrates into the tissue will also include T lymphocytes, which work in concert with tissue macrophages to form granulomas in an attempt to wall off the infection (34). Clinical animals in the present study had significantly greater amounts of macrophages and MAP burden, including intracellular MAP, as shown through IF and histochemical analysis. Positive correlations were observed between AF and granulomatous inflammation scores. Furthermore, positive correlations were also observed between number of macrophages present and both AF and granulomatous inflammation scores. The ability to control intracellular MAP replication and dissemination directly reflects whether or not the host will persevere or succumb to the infection.

Non-classical effects of vitamin D_3_ on immune system function during encounters with infectious pathogens such as *M. tb* in humans (7, 8) and bovine pathogens including *Mycobacterium bovis* (*M. bovis*) (14, 35) and *Streptococcus uberis* (*S. uberis*) (10, 36) have led researchers to investigate the impact of this important nutritional factor on bovine paratuberculosis. Our lab has previously reported that clinical cows have significantly reduced circulating levels of 25(OH)D_3_ (12, 13). A similar relationship between low circulating 25(OH)D_3_ and mycobacterial disease has been made in the case of bovine (37) and human tuberculosis (7, 38). Reduced serum 25(OH)D_3_ in cows with clinical paratuberculosis may be a result of the intestinal pathology impeding general nutrient absorption from the diet. This also correlates with the observed gradual weight loss as infection worsens and severe emaciation seen in severe cases. Tissue pathology and granulomatous inflammation can vary widely within each stage of disease, but typically correlates with progression of disease severity (24, 39).

Expression and activity of enzymes that function in vitamin D_3_ metabolism have not been extensively investigated in MAP infected cattle. In the present study, total tissue and intracellular expression of CYP24A1, the hydroxylase that inactivates both 25(OH)D_3_ and 1,25(OH)_2_D_3_, did not differ among cows in different stages of paratuberculosis. However, there was a significant reduction in CYP27B1 expression in the ileum of clinically infected animals compared to subclinical cows and JD- controls for both total tissue expression (*P* < 0.05) and intracellular macrophage expression. Reduced CYP27B1 expression in clinical cows may be correlated with the observed decrease in serum 25(OH)D_3_ from this group, as this analog of vitamin D_3_ is the ligand for the CYP27B1 hydroxylase. Pro-inflammatory IFN-γ expression as a result of Toll-like receptor 2/1 (TLR2/1) signaling has been shown to upregulate expression of CYP27B1 in human monocytes cultured in vitamin D sufficient serum (40). Investigation of macrophage phenotypes in the ileum of MAP infected cattle has shown significantly reduced IFN-γ expression in JD+ clinical cows (23), providing further evidence for a relationship between these two factors. Greater levels of *CYP27B1* gene expression in bovine ileocecal valve tissue has been observed in clinical cows compared to subclinicals and JD- controls (12), which contrasts results in the ileum for the present study. However, clinical cows had high levels of plasma IFN-γ production following MAP antigen activation, and the authors found a significant positive correlation between *IFNG* and *CYP27B1* expression (12). Increased mRNA expression of *CYP27B1* has also been previously described in human monocytes and macrophages activated by IFN-γ, TLR2/1, and TLR4 (41, 42). Additionally, ileal macrophages from cows with clinical paratuberculosis have shown significantly reduced IL-1β expression (23). In human macrophages, IL-1β plays a role in promoting CYP27B1 expression, in concert with IL-15, through a Th1 cell expansion/IFN-γ positive feedback loop following TLR2/1 activation (41, 43, 44). These studies collectively provide evidence for a link between pro-inflammatory responses and vitamin D metabolism, which may have implications for progression of JD in cattle.

Intact VDR signaling mechanisms are essential in regulating inflammatory responses to pathogens. Induction of VDR expression in *M. tb* infected human macrophages has been shown to be a result of TLR2 activation (7). Additionally, VDR knockout mice experience more severe inflammation and higher death rates following experimentally induced colitis (45). Studies have also shown that VDR expression in the colon of dairy cattle decreases during parturition and expression levels are related to development of milk fever in early lactation (46). Investigating VDR expression in the ileocecal valve from cattle naturally infected with MAP has shown no significant differences in expression between subclinical, clinical, and healthy cows (12), which align with observations in the present study.

Interruption of the endosomal pathway and phagosome-lysosome fusion have been a prominent finding in cases of infection with pathogenic mycobacterial species but have not been extensively detailed in cases of bovine paratuberculosis. We observed significantly less overall intracellular Rab5 expression in macrophages from clinical cows compared to JD- control and subclinical animals, which showed similar levels of expression. Furthermore, the amount of Rab5 observed to colocalize with MAP inside macrophages from subclinical animals was markedly reduced and significantly less than that of clinical cows. This observation aligns with animals in earlier stages of disease having less MAP burden, and consequently less tissue pathology, and suggests that a successful blockage of abundant Rab5 recruitment from early endosomes may be a means to allow more time for intracellular replication. Heavy MAP burden may overall inhibit Rab5 expression, but with increasing amounts of MAP a higher probability in successful trafficking through the macrophage may be achieved. Reduced recruitment of Rab5 to *M. tb* containing phagosomes has been previously reported through its utilization of cell wall glycolipid ManLAM to block trafficking activity of early endosome autoantigen 1 (EEA1) by phosphatidylinositol 3-phosphate (PI3P) (3, 5). EEA1 is responsible for binding to and tethering of Rab5 and the subsequent fusion of the early endosome with the phagosome (47, 48).

No significant differences in overall Rab7 expression within ileal macrophages were observed in the present study; however, the greater amount of Rab7 associated with intracellular MAP in clinical cows support the idea that higher MAP burden, and perhaps more extracellular MAP, results in less efficiency of immune evasion. Notably, no detection of Rab7 and MAP colocalization was made in subclinical cows, providing further evidence that a feature of earlier stages of paratuberculosis is to be more efficient in evading the immune system in order to establish and maintain a chronic subclinical phase. Previous work in J774 macrophages showed that infection with live MAP results in reduced acquisition of late endosomal marker LAMP-2, inhibited phagosome-lysosome fusion, and higher pH values for MAP-containing compartments compared to those with dead MAP or non-pathogenic mycobacteria (2). Additionally, IFN-γ/LPS-treated J774 macrophages infected with live MAP had higher incidence of colocalization with late endosomal marker LAMP-1 and less bacterial survival compared to untreated macrophages (49). Other mycobacterial species have been shown to persist intracellularly through their retention in early endosomal compartments that retain Rab5 but inhibit acquisition of later effectors (50–52). Another report using a HeLa cell model has shown phagosomes containing *M. tb* acquire late endosomal Rab7, although subsequent maturation steps are abrogated (53). Furthermore, *M. bovis* BCG containing phagosomes can acquire Rab7, but the bacteria inhibits association with Rab7-interacting lysosomal protein (RILP) which is necessary for the phagosome's fusion with lysosomes (54).

The present study provides foundational insight into vitamin D_3_ signaling and endosomal trafficking within macrophages from cattle with paratuberculosis; however, further investigation is warranted to fully understand the dynamics of these pathways at each stage of disease. A more expansive panel of early and late endosomal markers would provide further detail for which steps in the trafficking pathway MAP employs virulence factors to block activity. Macrophage phenotype markers and cytokine expression would be a valuable addition to assess the efficiency of the macrophage's antimycobacterial functions within the intestinal tissue.

## Data availability statement

The raw data supporting the conclusions of this article will be made available by the authors, without undue reservation.

## Ethics statement

The animal study was reviewed and approved by National Animal Disease Center Animal Care and Use Committee.

## Author contributions

Experimental design was conceived by JS and TW. MH contributed to fluorescence protocol optimization. Experiments and data analysis were performed and first draft manuscript was prepared by TW. Image acquisition was performed by AS and TW. All authors contributed to manuscript revisions and read and approved the manuscript for submission.
